# Exploring the microbial diversity in Jordanian hot springs by comparative metagenomic analysis

**DOI:** 10.1002/mbo3.521

**Published:** 2017-08-10

**Authors:** Emad I. Hussein, Jacob H. Jacob, Muhamad Ali K. Shakhatreh, Mutaz A. Abd Al‐razaq, Abdul‐salam F. Juhmani, Christopher T. Cornelison

**Affiliations:** ^1^ Department of Biological Sciences Yarmouk University Irbid Jordan; ^2^ Department of Biological Sciences Al al‐Bayt University Al‐Mafraq Jordan; ^3^ Department of Medical Laboratory Sciences Jordan University of Science and Technology Irbid Jordan; ^4^ Department of Environmental Science Ca’ Foscari University of Venice Venice Italy; ^5^ Division of Research and Advanced Studies Kennesaw State University Kennesaw GA USA

**Keywords:** Afra, hot springs, Ma'in, metagenomics, microbial diversity

## Abstract

A culture‐independent approach was utilized in this study to reveal the microbial diversity in Jordanian hot springs represented by Ma'in and Afra hot springs. Water samples from Ma'in and Afra hot springs were collected in June 2015. The *in situ* temperature of water samples range was 38–59°C and the pH range was 7.4–8.4. The metagenome was extracted and analyzed using the next generation technology (bTEFAP ^®^). A total of 314,310 sequences were parsed and 288,452 were then clustered. The sequences were predominated by bacteria (>84%) and the relative abundance of archaea in each sample was <1%. Eukaryotic microorganisms were detected but with varying abundances (0.6%–15%). Because most of the detected sequences were found to belong to the domain of bacteria (196,936 sequences out 288,452), the bacterial sequences were utilized for further microbial analyses. With respect to alpha and beta diversity, samples were rarefied to 30,000 sequences and bootstrapped at 10,000 sequences. The Shannon–Wiener Index curve plot reaches a plateau at approximately 3,000 sequences indicating that sequencing depth was sufficient to capture the full scope of microbial diversity. By examining the relative abundance of phyla detected in each sample, it appears that the biota of both Jordanian hot springs sampled are compositionally similar, with over 50% of the microbial community of each sample being comprised of the phylum Proteobacteria. The second most abundant phylum was the phylum Bacteroidetes which represents more than 13% in each sample. The phylum Firmicutes was also detected with a significant abundance. However, lower abundance of *Deinococcus*, Verrucomicrobia, Planctomycetes, and Chloroflexi was detected. A principal coordinate analysis plot was generated based upon the weighted UniFrac distance matrix. By utilizing Monte Carlo simulations, we were able to determine that there were no significant differences in the microbial diversity between each sample.

## 1 INTRODUCTION

Hot springs are unique natural environments for thermophilic microorganisms. In the last decades, thermal environments and thermophiles have gained interest due to their scientific and biotechnological importance. For instance, studying of thermophiles is necessary for better understanding of the origin of life as many scientists believe that life might have arisen in high temperature, and in the evolution of life, there is evidence for thermophilic ancestors (Burgess, Wagner, & Wiegel, 2007). With respect to the biotechnological applications of thermophiles, the representative example in this field is the aerobic thermophilic bacterium *Thermus aquaticus*, which was isolated several decades ago from Yellowstone National Park (Brock & Freeze, 1969). *T. aquaticus* has become a source of Taq polymerase which has led for the development of polymerase chain reaction (PCR) (López‐López, Cerdán, & González‐Siso, 2013).

Hot springs are produced by the emergence of geothermally heated ground water in volcanically active regions (Burgess et al., 2007). Hot springs are found throughout the world but they are more concentrated in certain regions in the world. Hot springs vary widely in their temperature, chemical composition, and pH (Madigan, Martinko, Stahl, & Clark, 2009).

Jordan is among the countries known for having many hot springs that differ in their physicochemical properties. Among the well‐known Jordanian hot springs are Ma'in hot springs located in the middle region of the country between Madaba and Amman. Afra hot springs represent another site located in the south region of the country in Tafieleh governorate about 160 km south of Amman (Malkawi & Al‐Omari, 2010). Water temperature in Ma'in hot springs reaches about 63°C, whereas in Afra hot springs, the temperature reaches about 42°C (Al‐Batayneh, Jacob, & Hussein, 2011). From a geological point of view, Ma'in and Afra hot springs are related to the Dead Sea Rift, where several springs discharge hot water originating from the Lower Cretaceous Sandstone (Swarieh, 2000).

The microbial diversity of Jordanian hot springs represented by Ma'in and Afra hot springs was assessed by several researchers using culture‐dependent methods. Early studies have shown that Ma'in and Afra hot springs are populated by many thermophilic microorganisms belonging mainly to the domain of Bacteria and more precisely to the genus *Bacillus* (Elnasser, Maraqa, Owais, & Khraisat, 2006; Khalil, 2002; Khalil, Anfoka, & Bdour, 2003; Khalil, Salim, & Sallal, 1998; Malkawi & Al‐Omari, 2010). Lately, we have documented the isolation and characterization of two new thermophilic bacterial species belonging to the genera *Geobacillus* and *Anoxybacillus* from both Ma'in and Afra hot springs (Al‐Batayneh et al., 2011). Data from the aforementioned studies confirm that applying enrichment and isolation approach results in the isolation of limited number of species belonging to the bacterial genus *Bacillus* or *Bacillus*‐related species. However, it must be noted that most thermophilic microorganisms in hot springs are generally unculturable (Kemp & Aller, 2004). Subsequently, the microbial diversity using culture‐dependent methods seems to be underestimated in Jordanian hot springs.

New molecular methods using metagenomic techniques enable researchers to characterize microorganisms found in hot springs by extracting the total DNA which includes the genetic material of microorganisms that cannot be cultured (López‐López et al., 2013). Therefore, a culture‐independent approach was utilized in this study to reveal the microbial diversity in Jordanian hot springs represented by Ma'in and Afra hot springs. The metagenome was extracted from water samples and analyzed using the next generation technology (bTEFAP^®^), described first by Dowd, Sun, Wolcott, Domingo, and Carroll (2008) and have been used in describing the biota from different types of environmental samples.

## 2 MATERIALS AND METHODS

### 2.1 Water samples

Four water samples (1 L) from four Jordanian hot springs were collected in June 2015. Samples 1, 2, and 3 were collected from Ma'in hot springs located in a well‐known Jordanian touristic site found between Madaba and Amman in the middle part of Jordan. The *in situ* temperature of water ranged between 48 and 59°C and the pH between 7.44 and 7.76. The fourth sample (sample A) was collected from Afra hot springs located in Tafieleh governorate located about 160 km south of Amman. The *in situ* temperature of water was 38°C and the pH was 8.41. Table 1 shows the temperature, pH, and absolute location of the sample sources.

**Table 1:** Temperature, pH, and absolute location (latitude and longitude) of the studied hot springs

Sample	Source	Temperature (*in situ*)	pH (*in situ*)	Absolute location
Sample 1	Ma'in hot spring‐1	56°C	7.61	31° 36.50 N
				35° 36.814 E
Sample 2	Ma'in hot spring‐2	48°C	7.76	31° 36.505 N
				35° 36.811 E
Sample 3	Ma'in hot spring‐3	59°C	7.44	31° 36.519 N
				35° 36.708 E
Sample A	Afra hot spring	38°C	8.41	30° 58.083 N
				35° 38.538 E

### 2.2 Chemical analysis

The tested chemical properties of hot springs include total dissolved solids (TDS), electrical conductivity (EC), salinity, nitrate concentration, chloride ion concentration, and fluoride ion concentration. Chemical analysis was determined as mentioned previously by Hussein, Jacob, Jahmani, & Yousef, 2013 and Ta'any, Batayneh, & Jaradat, 2007.

### 2.3 DNA isolation

Water samples were filtered through 0.2‐μm membranes under vacuum. Cells‐containing membranes were then excised into pieces. Membrane pieces were then transferred to a sterile 50‐ml centrifuge tube. DNA extraction from water samples was then carried out using a E.Z.N.A^®^ Water DNA kit (Omega Biotech Ltd., India) according to manufacturer's instructions. The eluted DNA was stored at −20°C until use.

### 2.4 Metagenomic analysis

The metagenomes from water samples were analyzed by amplicon sequencing using next generation technology (bTEFAP^®^) (Dowd, Callaway, et al., 2008, Dowd, Sun, et al., 2008; Swanson et al., 2011; Eren et al., 2011). A reengineered modern versions of bTEFAP^®^ is now one of the important methods used for assessing microbiota, which has been adjusted to nonoptical sequencing technologies (for instance the Ion Torrent PGM, the Illumina MiSeq and HiSeq platforms). The 16S rRNA gene universal Eubacterial primers 515F GTGCCAGCMGCCGCGGTAA and 806R GGACTACHVGGGTWTCTAAT were used to analyze the microbial diversity in water samples on the Illumina MiSeq with methods via the bTEFAP^®^ DNA analysis service. A single‐phase 30 cycle PCR with a HotStarTaq Plus Master Mix Kit (Qiagen, Valencia, CA, USA) were carried out with these conditions: 94°C (3 min), then 28 cycles of 94°C (30 s); 53°C (40 s), and 72°C (1 min). The last elongation step was performed at 72°C (5 min). After PCR, all amplification products were mixed with equivalent concentrations and purified by Agencourt Ampure beads (Agencourt Bioscience Corporation, MA, USA). Samples were sequenced using the Illumina MiSeq chemistry per manufacturer's procedures.

The Q25 sequences obtained were treated using a proprietary analysis pipeline (http://www.mrdnalab.com, MR DNA, Shallowater, TX, USA). After that, sequences were denoised and chimeras were removed. After removal of singleton sequences, operational taxonomic units (OTUs) were defined, clustering at 97% similarity or 3% divergence. (Dowd, Callaway, et al., 2008, Dowd, Sun, et al., 2008; Edgar, 2010; Swanson et al., 2011; Eren et al., 2011). Then, the OTUs were taxonomically classified by BLASTn against a curated GreenGenes/RDP/NCBI‐derived database (DeSantis et al., 2006) andaccumulated into each taxonomic level into both “counts” and “percentage” files; where the “counts” files have the number of sequences, and the “percent” files have the relative percentage or proportion of sequences in each sample.

### 2.5 Statistical analysis

Statistical analysis was carried out by different computer packages (NCSS 2007, “R”, NCSS 2010 and XLstat). Analysis of alpha diversity was done as described earlier (Dowd, Callaway, et al., 2008, Dowd, Sun, et al., 2008; Edgar, 2010; Eren et al., 2011; Swanson et al., 2011) using Qiime (www.qiime.org). Significance is defined as *p* < .05.

### 2.6 Alpha diversity description

Alpha diversity is an ecology term that denotes to the diversity within a specific zone or ecosystem, and it is normally expressed by the number of species (i.e., species richness) in that zone or ecosystem. The number of OTUs at the species level was assessed to describe alpha diversity between the different groups. Alpha diversity essentially evaluates how many different bacterial species are within the given sample or treatment group.

### 2.7 Beta diversity description

Beta diversity is an analysis of the structure of bacterial community. This analysis is done by generating separate phylogenetic trees regardless of taxonomy for each sample then valuing each tree statistically. Then, a principal coordinate analysis is carried out to permit for visualization of 10 separate jackknife iterative comparisons. After that, the multidimensional space is defined within the three primary vectors. Beta diversity essentially allows comparison of the community of bacteria as a whole taking into account both how many different sequences are in the sample and what those sequences are related to phylogenetically.

## 3 RESULTS

### 3.1 Chemical analysis

The chemical properties of hot springs were tested. The tested properties include TDS, EC, salinity, nitrate concentration, chloride ion concentration, and fluoride ion concentration. The results of chemical analysis are shown in Table 2.

**Table 2:** Chemical properties (total dissolved solids [TDS], electrical conductivity [EC], salinity, nitrate [${\text{NO}}_{3}^{-}$] concentration, chloride ion [Cl^−^]concentration, and fluoride ion [F^−^] concentration) of hot springs water obtained from Ma'in hot springs (Samples 1, 2, and 3) and Afra hot springs (Sample A)

Sample	TDS (mg L^−1^)	EC (mS cm^−1^)	Salinity (ppm)	${\text{NO}}_{3}^{-}$ (mg L^−1^)	Cl^−^ (mg L^−1^)	F (mg L^−1^)
Sample 1	1,544	3.32	1.6	1.83	630	4.63
Sample 2	1,543	3.26	1.6	1.81	637	2.90
Sample 3	1,543	3.05	1.5	1.74	592	1.83
Sample A	1,430	2.89	1.4	1.47	532	4.54

### 3.2 Metagenomic analysis

**Figure 1:**
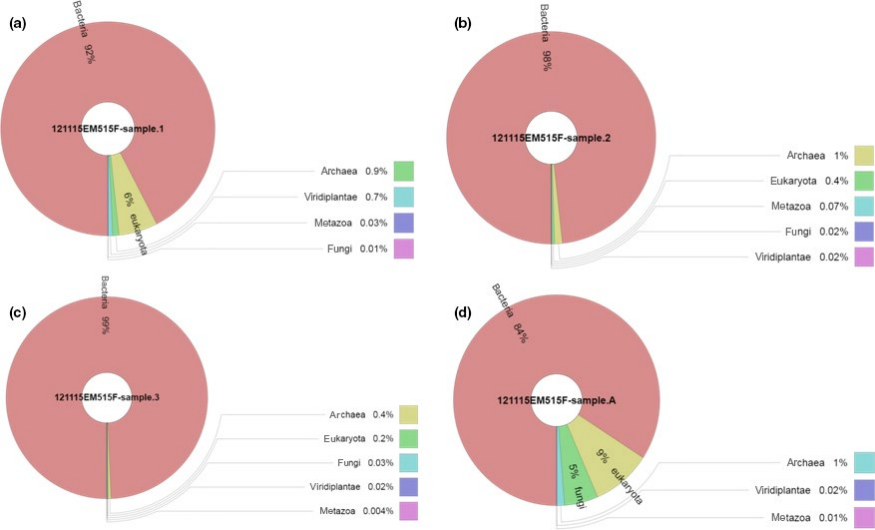
Relative abundance of microbial sequences detected in hot‐spring water samples (a, Sample 1; b, Sample 2; c, Sample 3; and d, Sample A)

After strict quality sequence curation, a total of 314,310 sequences were parsed and 288,452 were then clustered. The sequences were predominated by Bacteria (>84%). The relative abundance of bacteria in each sample was as following: sample 1 (92%), sample 2 (98%), sample 3 (99%), and sample A (84%). Very low abundance of archaea was detected in all samples (<1%). The relative abundance of archaea in each sample was as following: sample 1 (0.09%), sample 2 (1%), sample 3 (0.4%), and sample A (1%). In respect to eukaryotic microorganisms, very low abundance was also detected in sample 2 (1%) and sample 3 (0.6%). In respect to sample 1 and sample A, the abundance of eukaryotes was higher: sample 1 (7.1%), and sample A (15%). The abundance of different types of sequences is detailed and compared in Figure 1.

**Figure 2:**
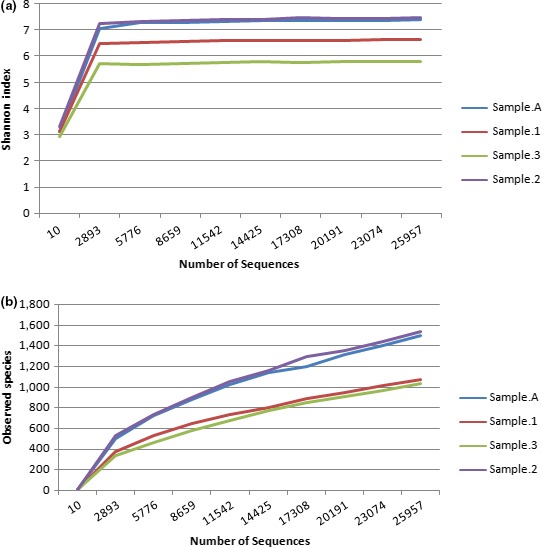
(a) Shannon–Wiener curve, and (b) rarefaction curve. Curves were calculated based upon 97% similarity

Because most of the detected sequences were found to belong to the domain of bacteria (196,936 sequences out 288,452), the bacterial sequences were utilized for further microbial analyses. The average reads per sample was 49,324. For alpha and beta diversity analysis, samples were refined to 30,000 sequences and bootstrapped at 10,000 sequences. The Shannon–Wiener Index curve plot (Figure 2) reaches a plateau at approximately 3,000 sequences indicating that sequencing depth was sufficient to capture the full scope of microbial diversity.

**Figure 3:**
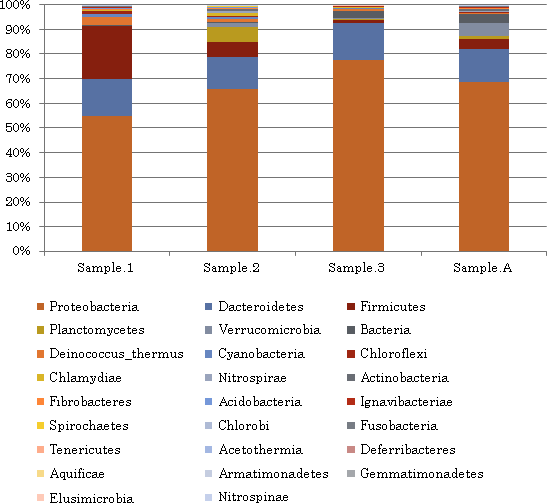
Relative abundance of bacterial phyla in Ma'in and Afra hot springs

In examining the relative abundance of phyla detected in each sample (Figure 3), it appears that the biota of both Jordanian hot springs sampled are compositionally similar, with over 50% of the microbial community of each sample being comprised of the phylum Proteobacteria: sample 1 (54.8%), sample 2 (65.8%), sample 3 (77.8%), and sample A (68.8%). The second abundant phylum was the phylum Bacteroidetes which represents more than 13% in each sample: sample 1 (15.2%), sample 2 (13.2%), sample 3 (15.0%), and sample A (13.4%). The phylum Firmicutes was also detected with a significant abundance. Analysis revealed that sample 1 does contain greater than three times the number of Firmicutes in comparison to the remaining samples. The relative abundance of Firmicutes in each sample was as following: sample 1 (21.5%), sample 2 (6.1%), sample 3 (0.1%), and sample A (4.1%). Lower abundance of *Deinococcus*, Verrucomicrobia, Planctomycetes, and Chloroflexi was also detected. The relative abundance of *Deinococcus* was 3.2% in sample 1, 1.5% in sample 2, and <1% in the rest of samples. The relative abundance of Verrucomicrobia was 1.8% in sample 2, 5.3% in sample A, and less than 1% in the rest of samples. The relative abundance of Planctomycetes was the highest in sample A (5.3%) and sample 2 (1.8%). Planctomycetes in the other samples is <1%. Finally, the relative abundance of Chlorofexi was the highest in sample 1 with 1.5% abundance and <1% in the rest of samples. Minor abundance of other phyla was also detected as shown in Figure 3.

**Figure 4:**
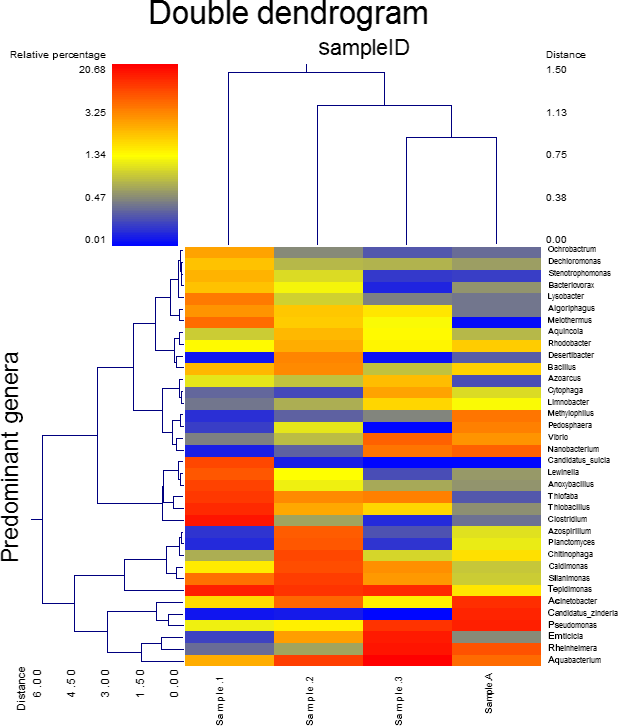
Dual Hierarchal dendrogram assessment of the taxonomic classification data. Samples that have similar microbial populations are clustered closer together. The genera or consortia are used here for clustering. Therefore, the samples with more similar genera or consortium cluster closer together and the length of connecting lines (top of heatmap) is correlated to the similarity. Shorter lines between two samples indicate closely matched microbial consortium. The heatmap denotes the relative percentages of each genus. The dominant genera are represented along the right *Y*‐axis. The heatmap legend is shown in the upper left corner

To provide a visual outline combined with analysis, we used a dual hierarchal dendrogram to show the data for the major genera with clustering related to the different groups. Based on the clustering evident in Figure 4, it appears the microbial composition of samples 2, 3, and A are more similarly related to each other than to that found in Sample 1.

### 3.3 Beta diversity of samples

**Figure 5:**
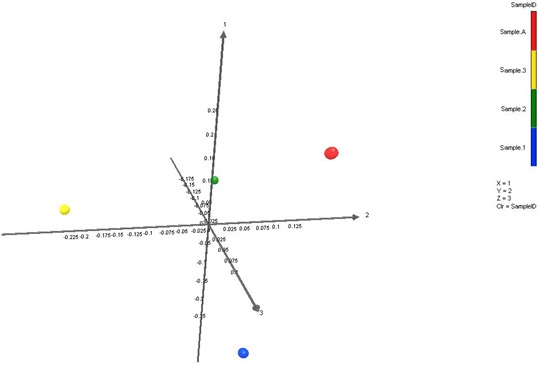
Principal coordinate plot of weighted Unifrac data with colors keyed on Sample 1(Blue), Sample 2 (Green), Sample 3 (Yellow), and Sample A (Red). Based on the primary vector which explained 48.5% of the variation between the samples, the first three vectors together exhibit 100% of the variation among the samples

A principal coordinate analysis plot was created based upon the weighted UniFrac distance matrix (Figure 5). By utilizing Monte Carlo simulations, we were able to determine that there are no significant differences in the microbial diversity between each sample (Table 3).

**Table 3:** Weighted UniFrac significance tests

Sample 1	Sample 2	*p* value (Bonferroni corrected)
Sample 1	Sample 2	1
Sample 1	Sample 3	1
Sample 1	Sample A	.372
Sample 2	Sample 3	1
Sample 2	Sample A	1
Sample 3	Sample A	1

UniFrac *p* values were based on comparisons to 1,000 randomized trees. The *p* values were only listed if they were >.05. All other pairwise comparisons indicated a significant difference between the samples.

## 4 DISCUSSION

The studied water samples were primarily differing in temperature and pH, whereas the other chemical properties were very close to each other. For instance, Ma'in hot springs are characterized by relatively high temperature (48–59°C) and neutral pH (7.44–7.76), whereas Afra hot springs are characterized by lower temperature (38°C) and slightly alkaline pH (8.41). With respect to the other chemical properties, high TDS level, Cl^−^ and F^−^ concentrations, and low EC and ${\text{NO}}_{3}^{-}$ concentrations were detected in all hot springs (as compared to standards of Environmental Protection Agency, 2012).

With respect to the microbiology of the studied springs, previous studies on Ma'in and Afra hot springs have mostly focused on microbial enrichment and isolation (Al‐Batayneh et al., 2011; Elnasser et al., 2006; Fandi, Al‐Muaikel, & Al‐Momani, 2014; Khalil et al., 2002; Khalil et al., 1998, 2003; Malkawi & Al‐Omari, 2010). Subsequently, many thermophilic bacteria were detected in Jordanian springs. Most of the isolated bacteria from the Ma'in hot springs belong to the bacterial genera *Bacillus*,* Geobacillus*, and *Anoxybacillus*. However, as expected, a greater diversity of microorganisms was detected by the culture‐independent, metagenomic approach. The current metagenomic analysis of Jordanian hot springs detected large number of bacterial phyla that were not previously described in the studied springs.

The obtained metagenomes indicate the dominance of bacteria. Surprisingly, archaea was found to be a minor group in the studied hot springs. When comparing our results with the results of other researchers, brings this finding into question. For example, 16S rRNA gene phylogenetic analysis of 10 hot springs in Tibet has revealed 959 sequences, 415 for bacteria and 544 for archaea, indicating that archaeal sequences are more abundant than bacterial sequences (Huang et al., 2011). In another study, Mardanove et al. (2011) had analyzed the thermophilic microbial community dwelling the groundwater at the East Thermal Field of Uzon Caldera, Kamchatka, and they had found that bacteria represent about 30% of microorganisms and more than 70% of microbial communities was represented by *Archaea*.

Within the dominant domain, i.e., bacteria, it was clear that the microbiomes of Jordanian hot springs compositionally similar, with over 50% of the microbial community of each sample being comprised of the phylum Proteobacteria. The second most abundant phylum is Bacteroidetes which represents more than 13% of each sample. The phylum Firmicutes was also detected with a significant abundance. Lower abundance of *Deinococcus*, Verrucomicrobia, Planctomycetes, and Chloroflexi were also detected. The prokaryotic diversity in Jordanian hot springs seems to be different from other hot springs in the world. For instance, the Bor Khlueng hot spring in Ratchaburi province, Thailand, was analyzed by a culture‐independent molecular approach. About 23% of the clones were found to belong to Acidobacteria. The other clones were classified as Bacteroidetes (19%), Nitrospirae (13%), Proteobacteria (12%), Deinococcus‐Thermus lineage (11%), Planctomycetes (6%), and Verrucomicrobia (5%). The four remaining phyla (5% each), were classified as Cyanobacteria*,* Chloroflexi, Actinobacteria, and the division “OP10”. (Kanokratana, Chanapan, Pootanakit, & Eurwilaichitr, 2004).

In another study, Sahoo, Subudhi, and Kumar (2015) studied samples from two hot springs (Atri and Taptapani) and found that Proteobacteria dominated the Taptapani sample metagenome (45.17%). The next most abundant phylum was Bacteroidetes (23.43%) and Cyanobacteria (10.48%). However, in the Atri sample, the most abundant phyla are Chloroflexi, Nitrospirae and Proteobacteria with 52%, 11%, and 10% dominance, respectively. Moreover, a large number of sequences remained taxonomically unknown.

This study highlights the unique ecology of Jordanian hot springs compared similar studies from other locations around the globe. As genomic databases become more complete, particularly regarding extremophiles and unculturable microbes, the taxonomic classification of currently unresolved sequences will strengthen the comparative analysis of metagenomic datasets, such as presented within this manuscript, and allow for a more complete understanding of these unique environments.

## CONFLICT OF INTEREST

The authors declare no conflict of interests regard the work presented in this manuscript.
